# Fukushima Outpatient Pharmacotherapy Model for Breast Cancer

**DOI:** 10.31662/jmaj.2024-0060

**Published:** 2024-09-06

**Authors:** Akihiko Ozaki, Ayaka Azami, Yusuke Azami, Tomomi Sugeno, Ayu Yasui, Kenji Gonda, Tetsuya Tanimoto, Kazunoshin Tachibana, Tohru Ohtake

**Affiliations:** 1 Department of Breast and Thyroid Surgery, Jyoban Hospital of Tokiwa Foundation, Iwaki, Japan; 2 Department of Breast Surgery, Minami-Tohoku Hospital, Koriyama, Japan; 3 Qol Pharmacy Koriyama Store, Koriyama, Japan; 4 Medical Governance Research Institute, Tokyo, Japan; 5 Department of Surgery, Fukushima Medical University, Iwaki, Japan

**Keywords:** Breast Neoplasms, Medical Oncology, Intersectoral Collaboration

## Abstract

The Fukushima Model of Outpatient Pharmacotherapy for Breast Cancer was developed to improve the pharmacological treatment of patients with breast cancer in the vast region of Fukushima Prefecture. This model addresses the challenges posed by the area’s lower-than-average density of breast cancer specialists. In the core medical institutions of the prefecture’s most populous municipalities, we introduced a telephone consultation service managed by pharmacists at local dispensing pharmacies. The novelty of the Fukushima model lies in two distinct elements: a structured checklist-style tracking report and comprehensive patient information sheets. This innovative tool streamlines a range of processes, including patient self-assessment of symptomatology associated with treatment-related side effects, subsequent medical interventions, and a standardized protocol for reporting severe side effects to healthcare practitioners. This approach facilitates the safe administration of breast cancer pharmacotherapy in home settings. In the future, this model could be used beyond Japan to underserved regions globally, thereby increasing the standard of breast cancer care on a wider scale.

## Introduction

Management of side effects during breast cancer therapy is of paramount importance. Recently, a substantial shift has been observed in the frequency of medical therapy sessions for breast cancer in outpatient settings. This transition offers benefits, particularly because many women play critical roles in their families and workplaces. However, this change has led to a reduction in medical oversight regarding side effects that occur in home settings.

To support patients with breast cancer, the role of pharmacists has been amplified in previous studies^(1), (2)^. Indeed, the role of oncology pharmacists has expanded over the last several decades ^(3)^. Oncology pharmacists, with their specialized training and technological advancements relieving them of dispensing duties, are now providing direct patient care alongside the healthcare team in clinical settings ^(3)^. They play a crucial role in delivering evidence-based cancer care, educating patients and caregivers on therapies, enhancing medication adherence, and informing healthcare team members about oncology drugs ^(3)^. Their clinical acumen and ability to evaluate medical literature enable them to contribute to the creation of guidelines and clinical pathways at various organizational levels, extending from institutional to global ^(3)^.

In Japan, breast cancer represents a crucial health challenge, with the number of cases on the rise; the number of new diagnoses in 2019 reached 97,812 ^(4)^. Concurrently, the range of treatment options has expanded, including the development of CDK4/6 inhibitors, PARP inhibitors, and immune checkpoint inhibitors. Reflecting on these developments, the Japan Oncology Drugs Market is projected to grow from a value of USD 13.68 billion in 2023 to an estimated USD 30.81 billion by 2030 ^(5)^. Amid these developments, the Japanese Society of Pharmaceutical Oncology recognizes pharmacists who have proven expertise in outpatient cancer chemotherapy with the title of Board-Certified Pharmacist in Ambulatory Cancer Chemotherapy ^(6)^. As of November 20, 2023, there are 757 pharmacists with this prestigious certification but only four in Fukushima ^(7)^. In a comparative international context, Japan is recognized for its substantial pharmacist workforce, boasting the highest ratio among the Organization for Economic Cooperation and Development countries, with 190 pharmacists per 100,000 population in 2019 ^(8)^. Nonetheless, the role of specialized pharmacists and their integration into clinical practice for breast cancer treatment is still not sufficiently documented in Japan.

## Fukushima Model of Outpatient Pharmacotherapy for Breast Cancer

This paper introduces the Fukushima Model of Outpatient Pharmacotherapy for Breast Cancer, which is currently being implemented to address the unique challenges in Fukushima Prefecture, Japan. Given the considerable size of the region, patients often experience difficulties in accessing medical facilities. The scarcity of specialists is notable, with only 0.20 breast cancer specialists per 100 km^2^ in Fukushima Prefecture compared with the national average of 0.54. This shortage has led to instances in which patients delay seeking medical advice despite recognizing the adverse symptoms, resulting in postponed diagnoses and improper use of supportive medications. A better coordinated approach among medical institutions, pharmacies, and patients could prevent these issues. To improve patient care access, the Minami-Tohoku Hospital in Koriyama City initiated a collaborative pharmaceutical program. This program is designed to streamline ambulatory breast cancer treatment and has followed successful initiatives in Kanazawa City, Ishikawa Prefecture. Additionally, it has been implemented in other areas of Fukushima, such as Iwaki City.

The Fukushima Model of Outpatient Pharmacotherapy for Breast Cancer is underpinned by documents called tracing reports, which report and provide feedback regarding patient adherence, medication residuals, side effects, and other relevant data. The tracing reports consist of three separate documents. The first document presents detailed patient information and treatment. The second document is intended for pharmacy use, enabling pharmacists to chronicle patient conditions following telephonic consultations. The third document is provided to the patients, who use it to facilitate and guide their responses during inquiries from the pharmacy.

Physicians issue tracing reports to patients in conjunction with prescriptions. Some of these reports, consisting of treatment documentation and pharmacist-directed forms, are presented to the pharmacists by the patients upon medication collection. The model advocates patient engagement via prearranged telephonic consultation with the pharmacy, which occurs approximately 1 week after the initiation of the medical therapy. The pharmacy calls the patients. Because the duration of chemotherapy for breast cancer is often longer than 1 week, we generally contact the patients on a weekly basis. During the consultation, pharmacists will identify any side effects requiring immediate clinical intervention. In establishing such interventions, pharmacists are mandated to communicate with relevant medical institutions and advise patients to seek urgent medical care. This mechanism is designed to facilitate timely access to medical interventions for patients.

This study highlights two distinct innovations in the Fukushima model. The first is a tailored checklist-style tracking report that streamlines several procedural aspects: it facilitates patient self-assessment of symptoms related to side effects, establishes criteria for medically significant side effects, and delineates the timing and methodology for pharmacist–patient communication. The guidelines also specify the protocols for reporting severe side effects to healthcare professionals. The second innovation is the use of patient information sheets. These sheets, which are disseminated to pharmacies, contain critical patient data, such as demographics, clinical conditions, primary complaints, and personalized chemotherapy regimens. This information equips pharmacists with the necessary knowledge to provide patient-specific pharmacological counseling. These sheets were primarily designed to reduce the workloads of healthcare professionals, such as physicians and nurses. Nonetheless, one strong point of this tool is that even pharmacists without special qualifications can safely administer chemotherapy.

**Figure 1.**
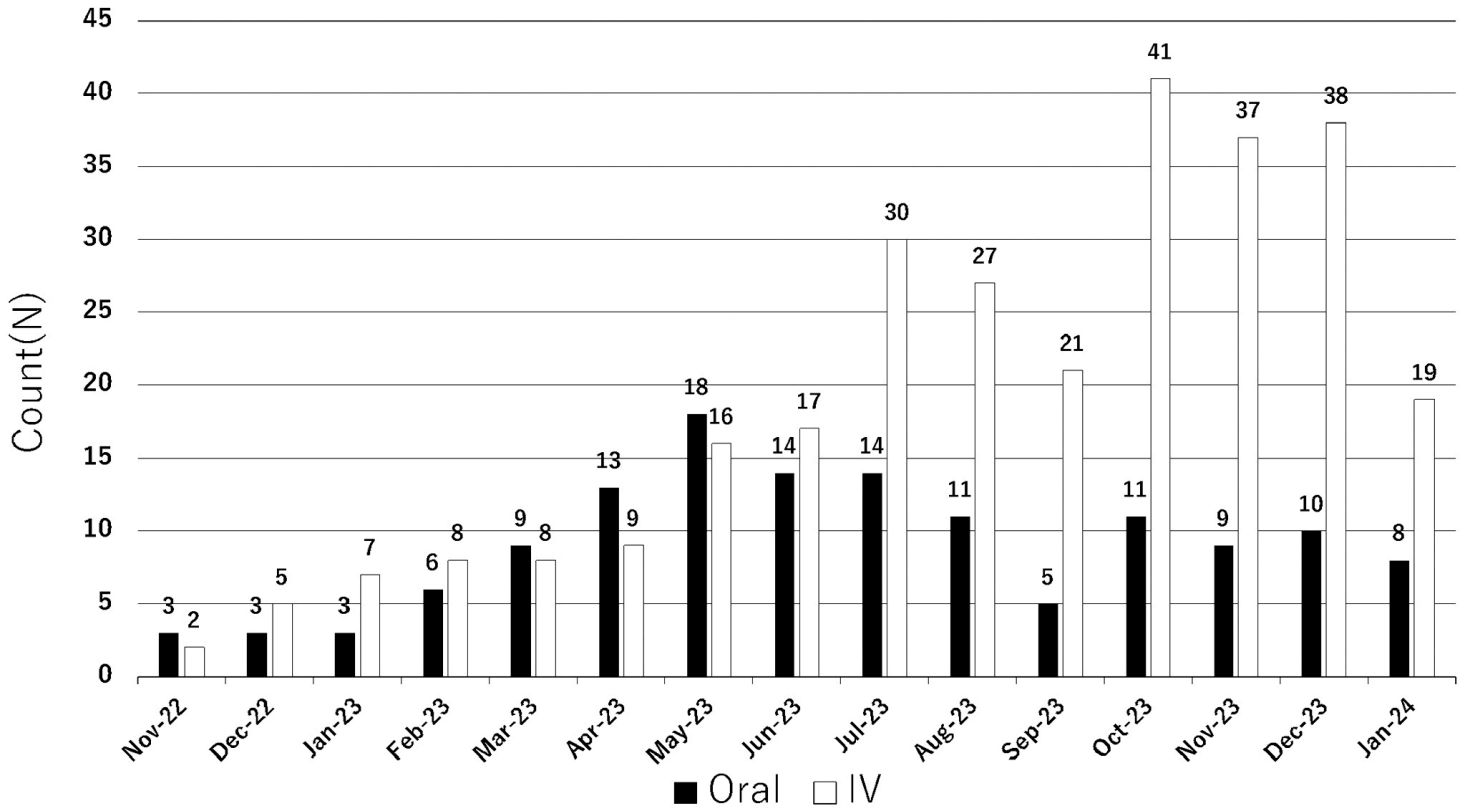
Monthly Distribution of Pharmaceutical Educational Sessions on Medical Therapies (November 2022–January 2024).

As depicted in Figure 1, the hospital implemented this model in November 2022 and offered support on 422 occasions until the conclusion of January 2024, without encountering significant issues. The details of the collaborative plans will be elaborated in forthcoming publications.

## Implications and Future Perspectives of the Fukushima Model

The initiation of this program transcends patient convenience and can substantively improve healthcare delivery. Despite the increasing incidence of breast cancer throughout Japan, disparities in healthcare professional allocation between urban and rural areas have intensified the plight of patients in medically underserved regions. Fukushima Prefecture is emblematic of such challenges, as the scarcity of medical resources necessitates innovative patient support approaches. The augmentation of pharmacotherapeutic options for breast cancer underscores the urgency for reinforced collaboration between hospitals and community pharmacies. Such partnerships are instrumental in ensuring a continuum of care, facilitating patient access to the latest treatments, and maintaining therapy adherence outside traditional hospital settings.

Furthermore, the scalability of the proposed model is promising for broad applications. Once refined and validated within the Japanese healthcare context, this logistical and collaborative framework can serve as a prototype for international adaptation. Considering global disparities in healthcare resources, the model’s principles can be tailored to fit diverse healthcare infrastructures, offering a strategic blueprint for improving oncological care delivery worldwide.

In some settings, sharing information via the Internet facilitates smoother coordination. However, there are many challenges associated with digital operations. For example, developing applications for digital information sharing is not feasible for hospitals in medically underserved areas because of a lack of funding. Moreover, elderly patients may find it challenging to report symptoms via the Internet because of skill limitations. Considering these reasons, paper-based operations are currently considered the best approach, at least in our setting. In regions where digital operations are feasible, developing suitable methods for such regions is desirable.

Future research should focus on the transferability of this model, assess its viability across different healthcare systems, and identify the necessary adjustments for global implementation. This research is crucial not only for extending the efficacy of successful healthcare innovations but also for promoting equity in global health outcomes.

In conclusion, we introduced the Fukushima Model of Outpatient Pharmacotherapy for Breast Cancer to improve the pharmacological treatment of patients with breast cancer in Fukushima Prefecture and more broadly address the challenges posed by the area’s lower-than-average density of breast cancer specialists. In the future, this model could be used beyond Japan to underserved regions globally, thereby increasing the standard of breast cancer care on a wider scale.

## Article Information

### Conflicts of Interest

Dr. Ozaki received personal fees from MNES; Kyowa Kirin Inc.; Becton, Dickinson and Company; Pfizer; and Taiho Pharmaceutical Co., Ltd., outside the scope of the submitted work.

### Author Contributions

Conceptualization: all authors; writing the original draft: Ozaki and Yasui; writing, review, and editing: all authors; visualization: Yasui; supervision: Tachibana and Ohtake.

### Approval by Institutional Review Board (IRB)

Not available.
